# Assessing the effect on the generation of environmentally persistent free radicals in hydrothermal carbonization of sewage sludge

**DOI:** 10.1038/s41598-019-53781-3

**Published:** 2019-11-19

**Authors:** Yuhan Zhu, Jia Wei, Yitao Liu, Xiaohui Liu, Jun Li, Jing Zhang

**Affiliations:** College of Architecture Engineering, Beijing University of Technology, 100 Pingleyuan, Chaoyang district, Beijing 100124 China

**Keywords:** Environmental sciences, Environmental social sciences

## Abstract

Environmentally persistent free radicals (EPFRs) have attracted increasing research interest in recent years. Herein, the generation of EPFRs during the hydrothermal carbonization of sewage sludge (SS) was studied. First, the surface morphology, functional groups, constituent elements and free radicals were characterized for a holistic description of the raw SS and the selected hydrochar obtained from hydrothermal carbonization of SS (SHC). Then, the impact of hydrothermal temperature, residence time and initial pH on the formation of EPFRs was explored in detail through the investigation of *g*-factors and intensities of EPFRs identified in SHC. The results have shown that the formation of EPFRs was affected by the factors mentioned above, in which the impact of temperature is the greatest. Two types of EPFRs were spotted in the hydrochar, oxygen-centered (O-centered) and carbon-centered (C-centered) EPFRs, which were caught in 120–150 °C and 260–280 °C, respectively. Moreover, the intensities of Electron Paramagnetic Resonance (EPR) signals enhanced with increasing hydrothermal temperature. Whereas, residence time and initial pH only affected the amount of EPFRs in a manner. Additionally, the half-life of the O-centered EPFRs and the C-centered EPFRs was determined as long as 160.45 days and 401.10 days, respectively, indicating that EPFRs are stable in a long time.

## Introduction

Nowadays, one of the most significant aims of environmental science is to treat and remove various contaminants. They originate from a number of different sources, which include, but are not limited to agricultural and industrial activities, human settlements and resource uses, etc. Primary pollutants are directly emitted into the environment as a result of the above processes, and some toxic secondary pollutants are further formed as a result of chemical reactions of these molecules in the environment. So far, hundreds of these contaminants have been studied in detail and regulated on basis of their well-known toxicity and/or other negative environmental impacts. However, a current trend of environmental emphasis is the so-called “emerging pollutants”, defined as newly identified or previously unrecognized contaminants^1^ (e.g., perfluorinated compounds (PFCs)^2^, pharmaceuticals and personal care products (PPCPs)^3^ and chlorination disinfection by-products (CDBPs)^4^). Environmentally persistent free radicals (EPFRs) are a new class of emerging pollutants^5^ which have attracted the attention of scientists increasingly. They are environmental chemical substances with strong durability, relatively low reactivity and serious environmental pollution and toxicity, owing to their potential of inducing the formation of biologically damaging reactive oxygen species (ROS) in biological systems^6^. According to several reports, they adversely influence infant respiratory health^7^, enhance severity of influenza virus infection^8^ and cause pulmonary obstructive diseases^9^, cardiovascular dysfunction and probably cancer^10^.

EPFRs were initially spotted in the soil of tropical region due to the existence of rich transition metal element, long sunshine duration and strong ultraviolet radiation in this area, which created a hotbed for generating EPFRs^11^. Then, EPFRs were found to be readily formed and emitted from thermochemical conversion of matter. For example, EPFRs were formed in the particulate matter produced by burning wood and coal^12^, in the residue produced by various plastics combustion^13^ and in the fly ash from municipal solid waste incineration. The thermal reaction processes are accomplished within a few seconds, but formed EPFRs have a long life span, which can persist for tens of minutes to several hours or even longer^14,15^. Dellinger and co-workers^15^ methodically investigated the generation of EPFRs by the pyrolysis of organics in the presence of transition metals, and they found that EPFRs had existed 74 min. And even the EPFRs immobilized on particulate emissions during the pyrolysis of biodiesel had a half-life of 431 days^16^. Furthermore, EPFRs in the biomass charcoal obtained by the pyrolysis of various plant based biomass raw materials^17^, were proved to be different in the content. For example, Fang *et al*.^18^ discovered singlet EPR signal during the pyrolysis of pine needles. Liao *et al*.^19^ explored that the intensive EPFRs signals were observed with the raised temperature in the pyrolysis of corn stalks, rice and wheat straws. Therefore, it is likely that there are still many unrecognized sources and precursors for forming EPFRs, garnering considerable interest in the fields of environmental engineering and material science.

The sewage sludge (SS) is the byproduct during waste water treatment process, which contains high concentration of heavy metals, pathogenic bacteria, organic compounds as well as substantial biomass. Therefore, it is often landfilled or incinerated to reduce their environmental hazards. On the other hand, it also can be served as raw material for making some profitable biomass based carbon materials through pyrolysis or hydrothermal technique^20,21^. Hydrothermal carbonization technology is favored for preparing in benign conditions, being not affected by the moisture content of raw material, and reducing the energy consumption. However, it is worth noting that during the hydrothermal treatment of SS, EPFRs may be apt to be formed and stabilized in the obtained solid product (hydrochar) through the interaction of organics and heavy metals. Moreover, compared with pyrolysis method studied most in the past, hydrothermal carbonization is different in many ways. Thus, it’s far more likely that the amount and type of formed EPFRs are different. To the best of our knowledge, no studies have been conducted to assess the presence of EPFRs and their persistence and properties in the hydrothermal carbonization of SS, and thus critical information is missing for the evaluation of potential risks from SS based hydrochar which will restrict its resource utilization.

Here, we aim to fill such important information gaps by investigating the generation and persistence of EPFRs through the hydrothermal carbonization of SS under different hydrothermal conditions. The main objectives of the study were to: (1) characterize the formed EPFRs in hydrothermal carbonization of SS; (2) examine the influencing factors including hydrothermal temperature, residence time and initial pH on the formation of EPFRs in the SS based hydrochar (SHC); and (3) unveil the potential mechanism of formation and transformation of EPFRs during the hydrothermal process. Hence, this work will provide valuable information for the in-depth understanding of the environmental behavior of EPFRs and offer scientific theoretical guidance for the innocuity and stabilized treatment and resource utilization of SS.

## Materials and Methods

### Materials

In this study, SS collected from Gaobeidian Sewage Treatment Works (Beijing, China) was chosen as the raw material for hydrothermal carbonization. It was first centrifuged for 1 min at 5000 rpm, and then was dried naturally for 24 h by a FD-1-50 vacuum freeze dryer (−50 below centigrade (no load)). HCl and NaOH purchased from Beijing Chemical plant were mixed with the deionized water to make up a certain concentration of solution to adjust pH of pretreated SS.

### Preparation of SHC

The preparation of SHC was performed in a 1L autoclave reactor (CFJ-1L Zhengzhou North-South Instrument, Henan, China), which was controlled by PID (Piping and Instruments Diagram) controller. In each batch experiment, the pretreated SS (water content 92%) was loaded into the reactor at room temperature. After that, the reactor was sealed off and then the nitrogen with a purity of 99.999% was offered from a cylinder into the reactor in order to create an anaerobic environment circumstance. The added SS was then stirred at a speed of 180 rpm and heated from the room temperature to a designed temperature between 120 and 280 °C under self-generated pressure (0–5 MPa) for a predetermined period. Subsequently, the autoclave was kept at the final pre-set temperature for a certain residence time, which was defined as the time that the reactor was held at a desired reaction temperature, excluding preheating and cooling time. When the pressure and temperature dropped to the atmospheric pressure and room temperature, the solid residues were taken out to centrifuge and dried in the vacuum freeze dryer until constant weigh. Finally, it was stored in the absence of air and placed in the refrigerator until further analysis. The obtained SHC was labeled as SHC_T-t-pH (Temperature-time-pH)_.

### Characterizations

Scanning electron microscopy (SEM) (JSM-6460LV Japan Electronics Corporation) was used to characterize the morphology of hydrochar samples with 10000 magnifications. The abundances of elemental C, H, O and N in unhydrolyzed SS and hydrochar samples were estimated using a Vario EL cube Elemental Analyzer (Elementar, Germany). Fourier transform infrared spectra (FTIR) was recorded on the freeze-dried material dispersed in KBr pellets by using a VERTEX 70V (German Brook) Fourier infrared spectrometer, and the spectra was scanned over the range of 400–4000 cm^−1^. X-ray photoelectron spectroscopy (XPS) patterns were recorded using thermo scientific escalab 250Xi (Thermo Corporation, USA) with a monochromatic Al-Ka source.

The emergence and the concentration of EPFRs were carried out by the EPR spectrometry (JEOL FA-200). Firstly, approximately 5 mg sample of hydrochar was loaded into a micropipette tip (0.9 mm in i.d., 1.1 mm in o.d., and 125 mm in length) and sealed with vacuum grease at one tip. The loaded hydrochar was continuously monitored for free radical signals on an EPR spectrometer with a single cavity at room temperature (adequate modulation amplitude of 100.00 kHz and microwave frequencies of 9051–9052 MHz). In order to minimize errors, all tubes were adjusted to the same position inside the cavity. Finally, radical concentrations were calculated using the integration of first derivative signal and comparison with Mn^2+^ standard. Mn^2+^ was used as a standard because of its similar spectral profile to the EPR signals detected in hydrochar. Simultaneously, the *g*-factor was computed by equipment software (Microsoft Office Excel and the WinEPR acquisition), which was a comprehensive line of software, allowing control of the Bruker EPR spectrometer, data-acquisition, automation routines, tuning, and calibration programs on a Windows-based PC.

## Results and Discussion

### The overall description of the samples

**Figure 1:**
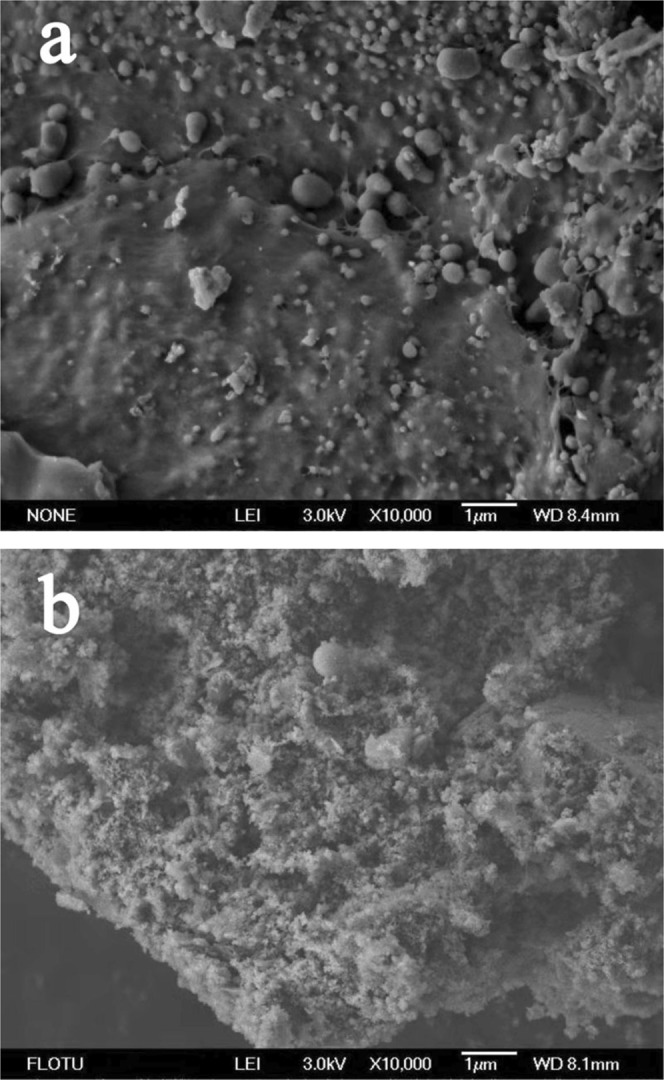
The SEM micrograph of unhydrolyzed SS (**a**) and SHC_180-2-7_ (**b**). (Images with 10000 magnifications).

A large number of sludge-based hydrothermal carbon samples were obtained by hydrothermal method. Initially, the unhydrolyzed SS and SHC_180-2-7_ are selected from scores of samples for a holistic depiction on the whole. As shown in Fig. 1, it is notable that the unhydrolyzed SS is planar and dense without pores and crevices. In contrast, the appearance of SHC_180-2-7_ presents flocculent structure, bearing an advanced pore network system. The contents of C, H, O and N in unhydrolyzed SS are 27.43%, 4.31%, 18.01% and 3.70%, respectively. It is found that C content (27.57%) does not change much after the hydrothermal carbonization, but H, O and N content all increase to 5.28%, 29.68% and 5.26% for SHC_180-2-7_.

**Figure 2:**
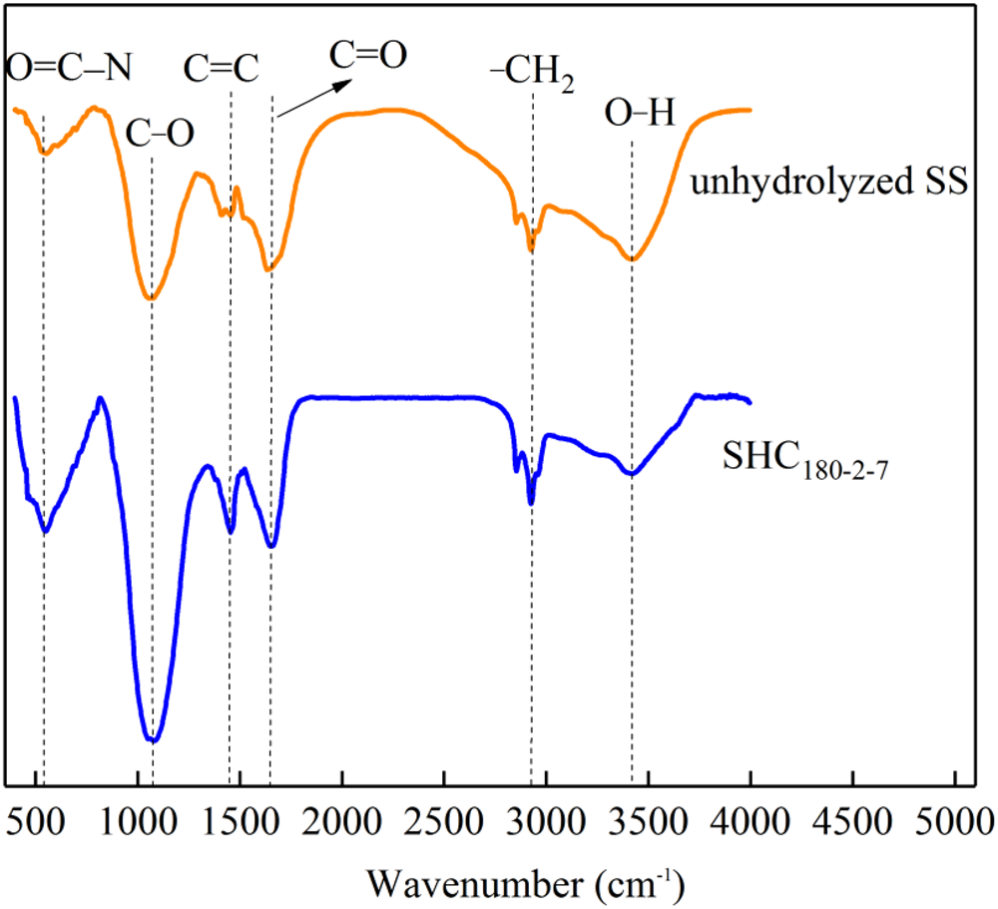
FTIR of unhydrolyzed SS and SHC_180-2-7_.

**Figure 3:**
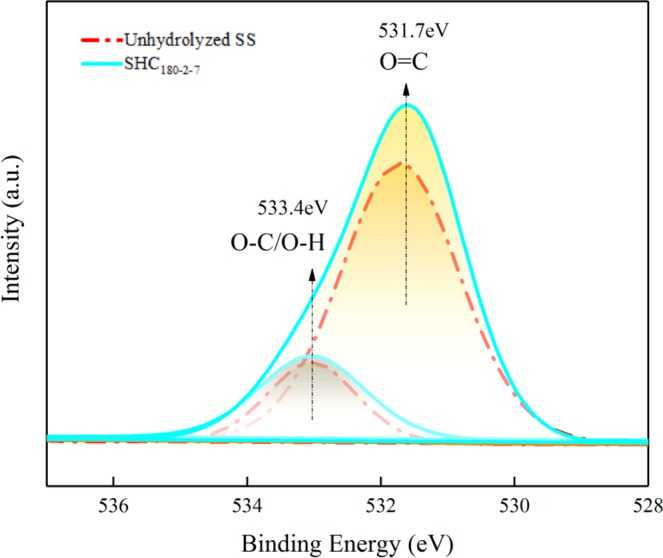
XPS of unhydrolyzed SS and SHC_180-2-7_.

As shown from the FTIR spectra of samples in Fig. 2, the peak existing between 3250 and 3600 cm^−1^ is ascribed to hydroxyl (–OH) stretching of the free COOH. The bands at 2922 cm^−1^ corresponds to methylene (–CH_2_) stretching vibration. The vibration modes at 1615 cm^−1^ is mainly ascribed to aldehyde (C=O), while the absorption band at 1460 cm^−1^ is reasonably assigned to alkene (C=C) in aromatic compounds. The intense broad absorption peak at 1056 cm^−1^ is characteristic of the C-O. The peak at 559 cm^−1^ is geared to the bending vibration absorption peak of O=C–N group which might be present in the SHC. It is distinctly realized from the FTIR spectra that the amount of surface functional groups of SHC raises, especially the C-O, O-H and C=O. This can be supported by the results of XPS (Fig. 3). 533.4 eV is attributed to O-C/O-H and 531.7 eV is described as O=C. It is true that the amounts of O-C/O-H and O=C increase demonstrated by the enhanced peak areas after hydrothermal carbonization.

**Figure 4:**
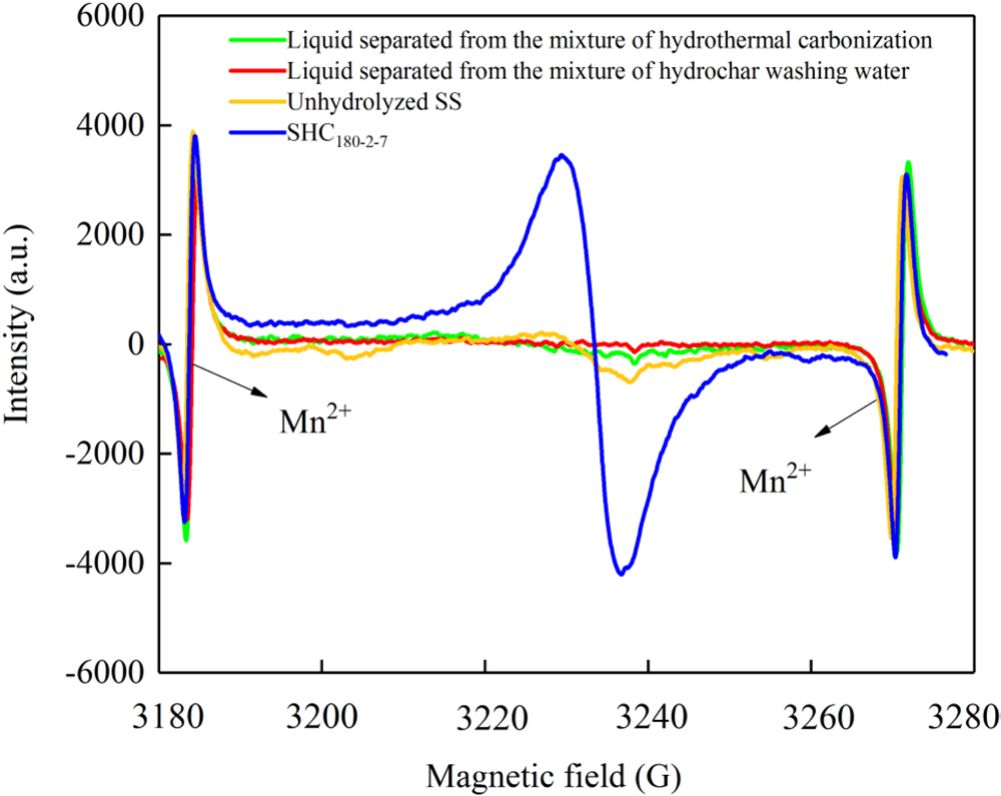
The EPR spectra of the selected samples.

As displayed in Fig. 4, the presence of EPFRs was studied by the EPR spectroscopy. The singlet EPR signal of SHC_180-2-7_ is obtained in the magnetic field of 3180–3280G, while weak signals are exhibited in the unhydrolyzed SS, two liquid samples separated from the mixture of hydrothermal carbonization and hydrochar washing water. The Mn^2+^ signal at both ends of the magnetic field is an internal manganese standard to measure simultaneously with the samples. The amount of EPFRs can be identified from the intensity or the peak area of samples. Here, the quantities of EPFRs are expressed by the peak area which is the integral of original EPR spectrum. The type of EPFRs in the SHC is identified through the *g*-factor computed from EPR signals. The *g*-factor, also known as the *g*-value, reflects the characteristics of the magnetic field in the molecule of matter^22^, which mainly depends on the strength of the coupling effect between spin motion and orbital motion. In addition, it determines the position of the spectral line in the EPR spectrum. When the matrix interaction or spin-orbit coupling effect^23^ changes the *g*-factor, the structure of EPFRs will change, signifying that new free radicals are formed in the system. Previous studies suggested that the *g*-factors less than 2.00300 were typical for (C-centered) free radicals^23,24^. Oxygen-centered (O-centered) free radicals usually possess the *g*-factor larger than 2.00400, such as semiquinone or phenoxyl radicals^25,26^. And radical signals with *g*-factors of 2.00300–2.00400 are attributed to a combination of the C-centered and O-centered EPFRs^26^. Obviously, the EPR spectrum of SHC_180-2-7_ in Fig. 4 shows a strong signal at the computed *g*-factor 2.00355, which confirms the generation of mixed C-centered and O-centered free radicals.

### The effect of hydrothermal temperature on the formation and transformation of EPFRs

Temperature has been deemed as one of the most momentous factors influencing hydrothermal carbonization of biomass^27^ which may also potentially affect the formation of EPFRs in the obtained hydrochar. Fig. S1 depicts the apparent EPR signals of all the SHC samples produced at different temperatures. It is clearly shown that the intensity of EPR signals raises with the increase of temperature from 120 to 280 °C. It was reported that EPFRs could be formed in biomass pyrolysis at high temperatures (200–600 °C)^18,28,29^. In comparison, EPFRs can be generated at relatively low temperature through the hydrothermal carbonization in this research, probably owing to the hydrolysis and cleavage of biomass by subcritical water^29^ which acts as an efficient solvent and catalyst. As temperature increases, it is anticipated that EPFRs can be formed from the cleavage of bonds in surficial functionalities of biomass^30^ or free radicals’ self-recombination.

**Figure 5:**
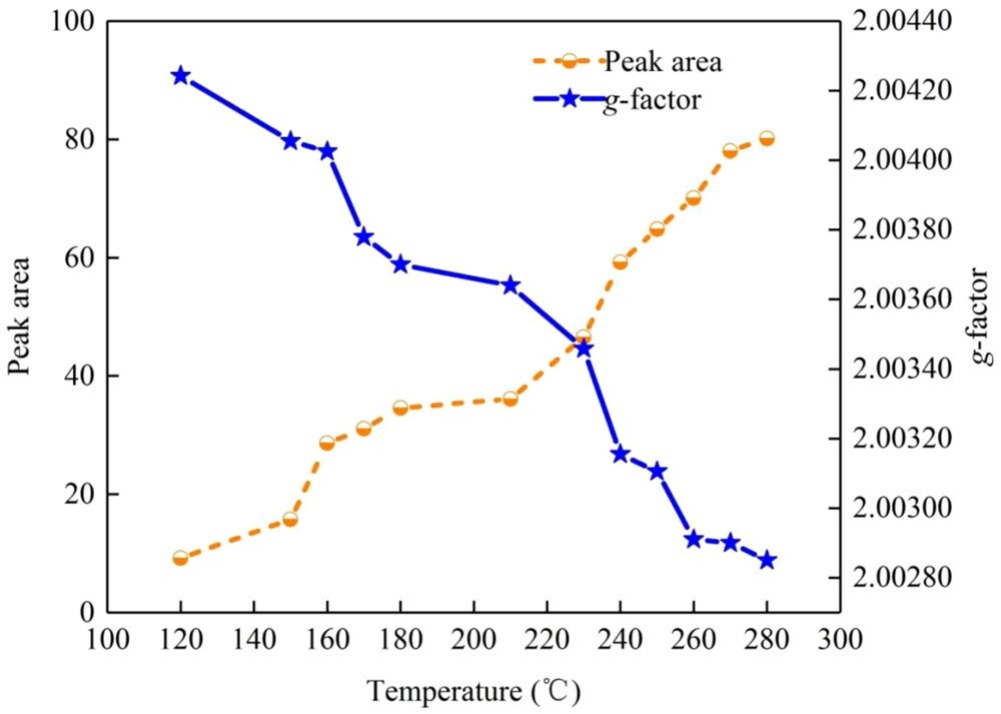
Peak area and *g*-factor of SHC prepared at different temperatures.

As shown in Fig. 5, the peak area of EPFRs obtained grows with the increase of temperature from 120 to 280 °C, while *g-*factor shows a diverse trend from the intensity of EPFRs. In our work, two types of radicals indicated by changed *g*-factors are observed. The *g*-factors decrease from 2.00415 to 2.00402 as the hydrothermal temperature increases from 120 to 150 °C, indicating that the dominant species are the O-centered EPFRs (e.g., aromatic radials). As the temperature raises from 150 to 250 °C, the C-centered EPFRs are prone to be formed under the high temperature, leading to a mixture of C- and O-centered EPFRs. Subsequently, with the raised temperature, the C-centered EPFRs increase in large numbers and finally become the dominant type of EPFRs confirmed by *g*-factors less than 2.00300 (Fig. 5).

**Scheme 1:**
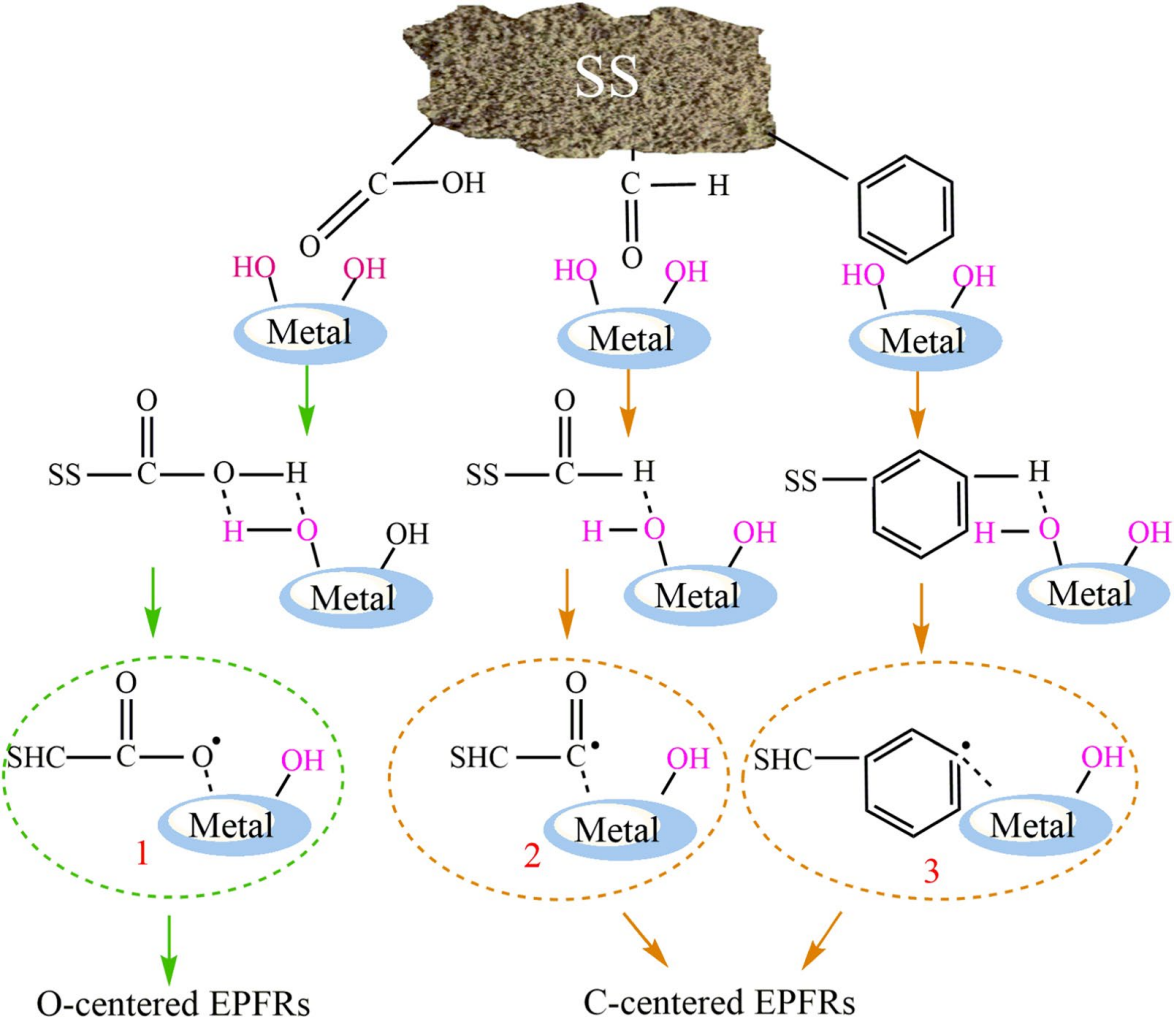
Proposed mechanistic pathways for the formation of O-centered and C–centered EPFRs from SHC.

As known to all, more than 50% to 80% of the heavy metals in the sewage enter into SS through sewage treatment. Undoubtedly, during the hydrothermal carbonization, organic components in SS may be partially decomposed or degraded and complex with transitional metals, forming EPFRs, which are stabilized in the system by “organic functional groups-biomass carbon particles”. The detailed possible channel for the formation of EPFRs in this study is presented in Scheme 1. There are a variety of functional groups on hydrochar, such as aldehyde (C=O), carboxyl (-COOH), aromatic and so on. O-centered EPFRs (radical 1) are obtained by the reaction of carboxyl group on SS with hydroxyl group on metal though elimination of H_2_O. Both aldehyde group and aromatic group bond chemically with hydroxyl groups on metal to form C-centered EPFRs (radical 2 and radical 3) via elimination of H_2_O in the process of hydrothermal carbonization. Similar results were reported that EPFRs formed by various precursor molecules on different metals^15,23,31^.

### The effect of residence time

**Figure 6:**
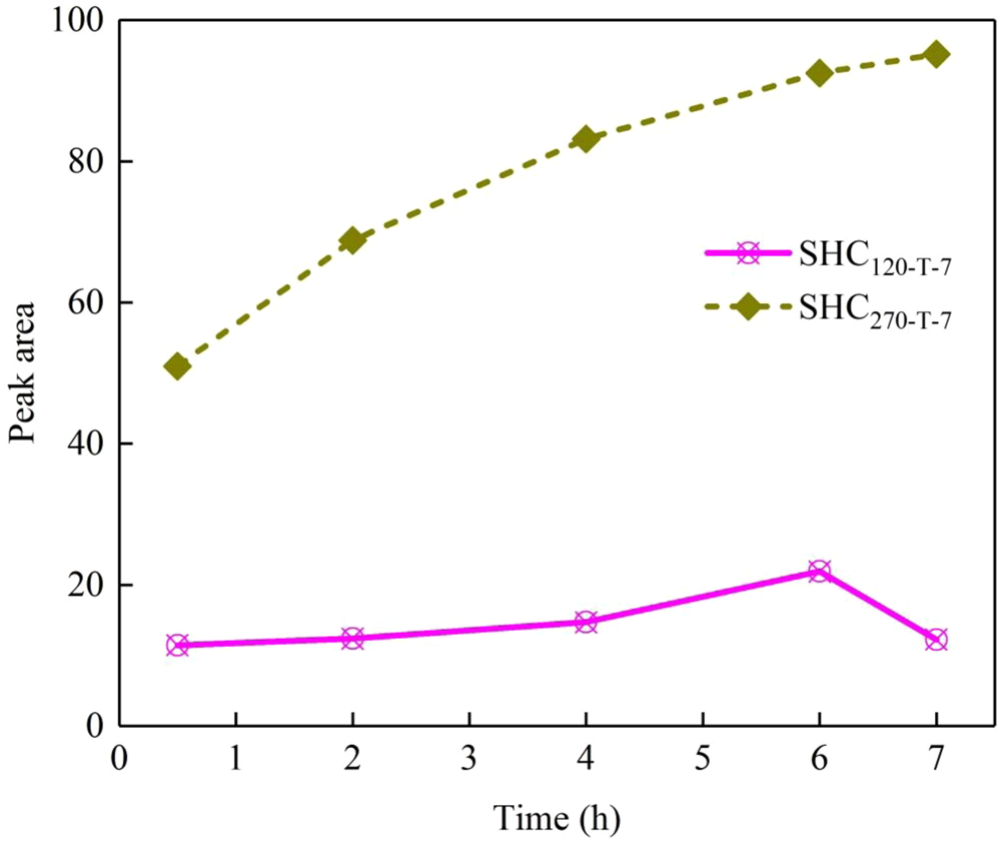
Peak area of the SHC prepared at 120 °C and 270 °C under different residence times.

It can be aware of the above results that two types of EPFRs are produced. The effect of hydrothermal residence time (0.5 h to 7 h) on the generation of EPFRs are respectively investigated under two temperatures, on which two types of EPFRs (O-centered and C-centered) are formed. The experiments under different residence time were conducted at pH = 7. As listed in Table 1, *g*-factors are all greater than 2.00400 for 120 °C and less than 2.00300 for 270 °C, meaning the types of EPFRs are not changed with the variation of residence time at 120 °C. However, the amounts of EPFRs exhibit significant changes with the prolonging residence time at 270 °C (Fig. S2). It is clearly seen from Fig. 6 that the O-centered EPFRs grow in number incrementally first and then decrease with the extension of residence time, acquiring optimum quantities in 6 h at 120 °C. The probable explanation is that the system needs a relatively long residence time to approach equilibrium of biomass conversion. Nevertheless, a prolonged period encourages the polymerization rearrangement bringing result that the formed EPFRs are decomposed and decayed. However, the quantities of C-centered EPFRs gradually increase in the range of setting hydrothermal conditions indicating the potential of producing more C-centered EPFRs with the extending time in hydrothermal carbonization of SS.

**Table 1:** The EPR Parameters (*g*-factors) of EPFRs in SHC at 120 °C and 270 °C under different residence times and different pH.

Time/h	t/°C		pH	t/°C	
	120	270		120	270
0.5	2.00417	2.00291	2	2.00460	2.00299
2	2.00425	2.00292	4	2.00440	2.00298
4	2.00447	2.00297	6	2.00416	2.00297
6	2.00441	2.00293	8	2.00406	2.00296
7	2.00421	2.00290	10	2.00408	2.00295
			12	2.00413	2.00293

^a^The experiments under different residence time were conducted at pH = 7.

^b^The effect of initial pH on EPFRs was experimented under 2 h residence time.

### The effect of initial pH

**Figure 7:**
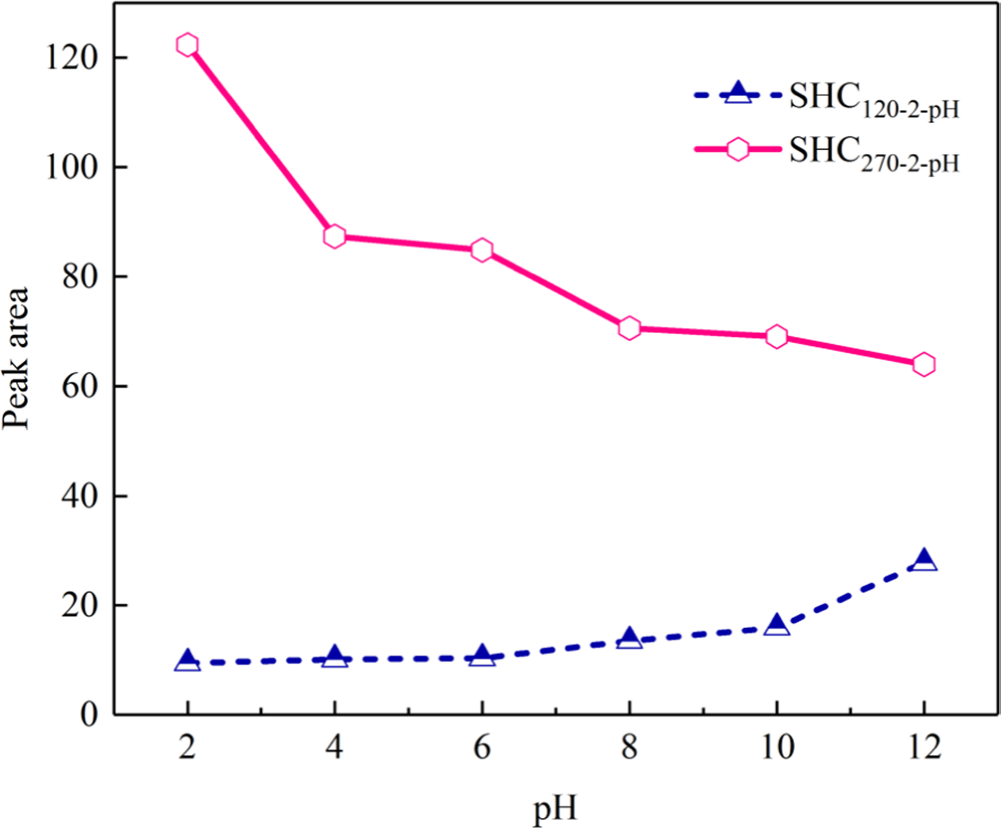
Peak area of the SHC prepared at 120 °C and 270 °C under different pH.

The O-centered and C-centered EPFRs produced at 120 and 270 °C are selected for investigating pH, respectively (Fig. S3). The effect of initial pH on EPFRs was experimented under 2 h residence time. Table 1 displays that the *g*-factors of SHC prepared under different pH change from 2.00406 to 2.00460 at 120 °C (O-centered EPFRs), and from 2.00293 to 2.00299 at 270 °C (C-centered EPFRs). The variations of *g*-factors with pH are similar with the influence of residence time that the type of EPFRs is only depended on the temperature. As indicated in Fig. 7, the yields of O-centered EPFRs exhibit a slow upward trend with the increase of pH at 120 °C. Because probably in the relative high pH environment, the oxygen containing functional groups of SHC tend to be reacted with the metals in high valence which are carried originally in SS^18^. However, the peak area decreases markedly from 122.383 to 63.946 at 270 °C with enhanced pH. Obviously, a relatively high acidity is potentially expected to produce more C-centered EPFRs from the cleavage of bonds in the hydrochar polymers at comparatively high temperature. Furthermore, the reduction of signal is ascribed to the transportation of electrons between C-centered EPFRs and metals. Thus initial pH is also regarded as a crucial parameter affecting free radical.

### Half-life of EPFRs

Generally, common free radicals have very short half-life within a range of 10^−9^–10^−4^ s^32^. However, in the event that the free radicals are generated near the solid particle surface, they may have strong interactions with the particles and exist steadily and permanently.

The dynamic behavior monitored over a period of 17 weeks was conducted to determine the persistence of the EPFRs in SHC particulates. The EPR test intervals were conducted 3 weeks (1.8 × 10^6^ s), 4 weeks (3.6 × 10^6^ s), 5 weeks (1.8 × 10^6^ s) and 6 weeks (1.8 × 10^6^ s), respectively. A pseudo-first order reaction kinetic model^16^ is adopted in which the global Eq. 1

1 $${\rm{lnC}}={{\rm{lnC}}}_{{\rm{0}}}-k{\rm{t}}$$

where constant *k* is found from the slope of the correlation between logarithm of EPFRs concentration change, C and C_0_ are the initial and tested concentrations of EPFRs, respectively, and t is tested time.

**Figure 8:**
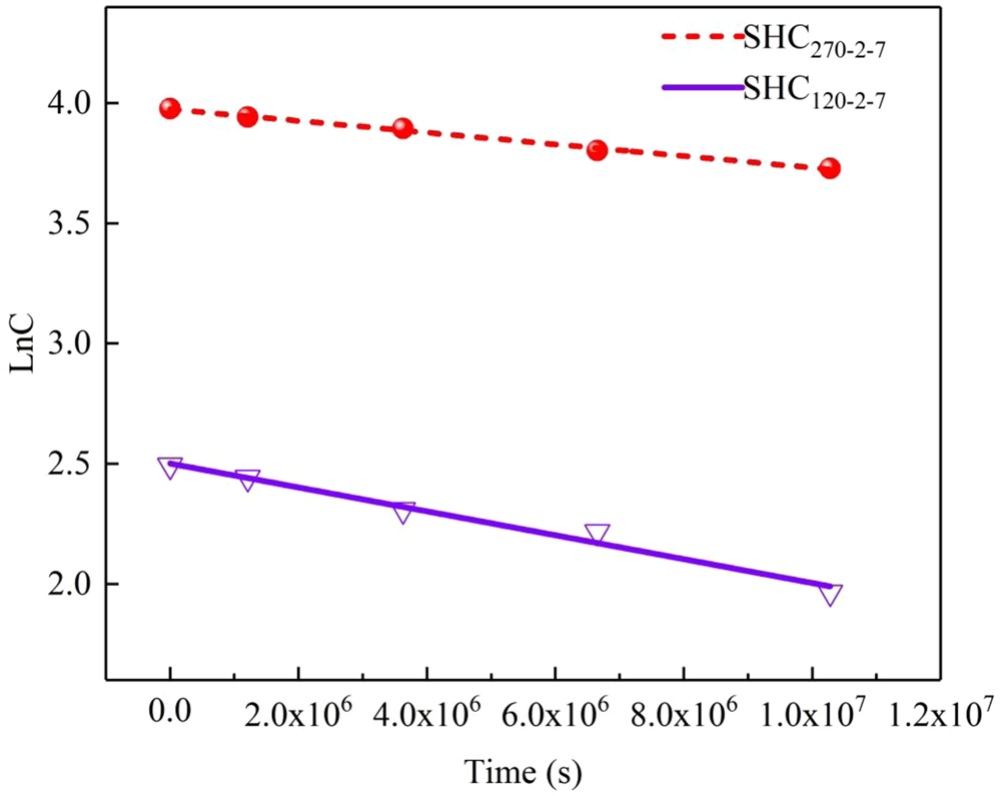
First-order decay (normalized) and half-life times of EPR signals in SHC_120-2-7_ and SHC_270-2-7_.

As shown in Fig. 8, the function diagrams of lnC and time t (s) are generated from which the kinetic parameters rate constant *k* and the initial concentration (C_0_) of two types of EPFRs in the SHC are determined.

The general expression given in Eq. 2^16^ is used to calculate the half-life decay of the EPFRs.

2 $${t}_{1/2}=\frac{{\rm{ln2}}}{k}$$

The slope of the graph (Fig. 8) gives the free radical rates constant *k* as 6 × 10^−8^ s^−1^ for SHC_120-2-7_, and 2 × 10^−8^ s^−1^ for SHC_270-2-7_. These rates constant are small, implying that the intensities of the radicals change little over the entire period. Then the calculated *k* constants are substituted in Eq. 2 to determine the half-life of two types EPFRs, respectively. The EPR signals of SHC_120-2-7_ exhibit a slow decay with a half-life of 160.45 days, indicating that O-centered EPFRs are relatively steady. Meanwhile, the C-centered EPFRs bear a half-life of 401.10 days which is even two times longer than O-centered EPFRs. In general, both of the O-centered and the C-centered EPFRs identified in the prepared SHC are able to persist in environment for a long time.

## Conclusions

Here, we make a thorough investigation about the generation and the persistence of EPFRs. The results have shown that the formation of EPFRs is depended on temperature, residence time and initial pH, while the type of EPFRs is only determined by temperature during the hydrothermal carbonization of SS. The O-centered EPFRs are apt to form under a relative low temperature. The main cause is that the oxygen containing groups transform into EPFRs via the catalysis of subcritical water. With the change of residence time, the yield of O-centered EPFRs increases originally and then decreases, yet the amount rises with the increased initial pH. High temperature is favorable for the generation of C-centered EPFRs. The longer residence time is, the larger the amount of C-centered EPFRs is. In addition, acidic condition is more conducive to the production of C-centered EPFRs. Furthermore, all radicals exit in the obtained SHC have long half-life. On the whole, temperature is the main factor controlling the type and quantity of EPFRs, but residence time and pH also affect them to a certain extent.

## Supplementary information

Supplementary information

## Data Availability

We declared that materials described in the manuscript, including all relevant raw data, will be freely available to any scientist wishing to use them for non-commercial purposes, without breaching participant confidentiality.

## References

[1] Magi E, Carro MD (2018). Marine environment pollution: The contribution of mass spectrometry to the study of seawater. Mass Spectrom. Rev..

[2] Weiss-Errico MJ, O’Shea KE (2017). Detailed NMR investigation of cyclodextrin-perfluorinated surfactant interactions in aqueous media. J. Hazard. Mater..

[3] Chen WL, Cheng JY, Lin XQ (2018). Systematic screening and identification of the chlorinated transformation products of aromatic pharmaceuticals and personal care products using high-resolution mass spectrometry. Sci. Total Environ..

[4] Do MT (2005). Chlorination disinfection by-products and pancreatic cancer risk. Environ. Health Perspect..

[5] Vejerano EP, Rao GY, Khachatryan L, Cormier SA, Lomnicki S (2018). Environmentally persistent free radicals: Insights on a new class of pollutants. Environ. Sci. Technol..

[6] Dellinger B (2001). Role of free radicals in the toxicity of airborne fine particulate matter. Chem. Res. Toxicol..

[7] Saravia J, Lee GI, Lomnicki S, Dellinger B, Cormier SA (2013). Particulate matter containing environmentally persistent free radicals and adverse infant respiratory health effects: a review. J. Biochem. Mol. Toxicol..

[8] Lee GI (2014). Exposure to combustion generated environmentally persistent free radicals enhances severity of influenza virus infection. Part. Fibre Toxicol..

[9] Gehling W, Khachatryan L, Dellinger B (2014). Hydroxyl radical generation from environmentally persistent free radicals (EPFRs) in PM2.5. Environ. Sci. Technol..

[10] Khachatryan L, Vejerano E, Lomnicki S, Dellinger B (2011). Environmentally persistent free radicals (EPFRs). 1. Generation of reactive oxygen species in aqueous solutions. Environ. Sci. Technol..

[11] Li FB, Wang XG, Zhou SG, Liu CS (2006). Reviews on abiotic transformation of organchlorines on the interface of iron oxides and water in red soil colloids *Ecol*. Environ..

[12] Cruz ALNd, Gehling W, Lomnicki S, Cook R, Dellinger B (2011). Detection of environmentally persistent free radicals at a superfund wood treating site. Environ. Sci. Technol..

[13] Valavanidis A, Iliopoulos N, Gotsis G, Fiotakis K (2008). Persistent free radicals, heavy metals and PAHs generated in particulate soot emissions and residue ash from controlled combustion of common types of plastic. J. Hazard. Mater..

[14] Cormier SA, Lomnicki S, Backes W, Dellinger B (2006). Origin and health impacts of emissions of toxic by-products and fine particles from combustion and thermal treatment of hazardous wastes and materials. Environ. Health Perspect..

[15] Lomnicki S, Truong H, Vejerano E, Dellinger B (2008). Copper oxide-based model of persistent free radical formation on combustion-derived particulate matter. Environ. Sci. Technol..

[16] Mosonik BC, Kibet JK, Ngari SM, Nyamori VO (2018). Environmentally persistent free radicals and particulate emissions from the thermal degradation of Croton megalocarpus biodiesel. Environ. Sci. Pollut. Res..

[17] Trubetskaya A (2016). Characterization of free radicals by electron spin resonance spectroscopy in biochars from pyrolysis at high heating rates and at high temperatures. Biomass Bioenerg..

[18] Fang GG, Liu C, Gao J, Dionysiou DD, Zhou DM (2015). Manipulation of persistent free radicals in biochar to activate persulfate for contaminant degradation. Environ. Sci. Technol..

[19] Liao SH, Pan B, Li H, Zhang D, Xing BS (2014). Detecting free radicals in biochars and determining their ability to inhibit the germination and growth of corn, wheat and rice seedlings. Environ. Sci. Technol..

[20] Lin YM, Zhou SQ, Li FZ, Lin YX (2012). Utilization of municipal sewage sludge as additives for the production of eco-cement. J. Hazard. Mater..

[21] Wei J, Liu YT, Li J, Yu H, Peng YZ (2018). Removal of organic contaminant by municipal sewage sludge-derived hydrochar: kinetics, thermodynamics and mechanisms. Water Sci. Technol..

[22] Valentin CD (2006). Density-functional model cluster studies of EPR g tensors of Fs^+^ centers on the surface of MgO. J. Chem. Phys..

[23] Vejerano E, Lomnicki S, Dellinger B (2011). Formation and stabilization of combustion-generated environmentally persistent free radicals on an Fe(III)_2_O_3_ silica surface. Environ. Sci. Technol..

[24] Fang GG, Zhu CY, Dionysiou DD, Gao J, Zhou DM (2015). Mechanism of hydroxyl radical generation from biochar suspensions: Implications to diethyl phthalate degradation. Bioresour. Technol..

[25] Jia HZ (2018). Transformation of polycyclic aromatic hydrocarbons and formation of environmentally persistent free radicals on modified montmorillonite: The role of surface metal ions and polycyclic aromatic hydrocarbon molecular properties. Environ. Sci. Technol..

[26] Jezierski A, Skrzypek G, Jezierski P, Paul D, Jedrysek MO (2008). Electron paramagnetic resonance (EPR) and stable isotope records of paleoenvironmental conditions during peat formation. Spectrochim. Acta A..

[27] Axel F, Felix Z (2010). Hydrothermal carbonization of biomass: A summary and discussion of chemical mechanisms for process engineering. Biofuel. Bioprod. Bior..

[28] Kibet J, Khachatryan L, Dellinger B (2012). Molecular products and radicals from pyrolysis of lignin. Environ. Sci. Technol..

[29] Mçller M, Nilges P, Harnisch F, Schrçder U (2011). Subcritical water as reaction environment: fundamentals of hydrothermal biomass transformation. ChemSusChem.

[30] Demirbaş A (2000). Mechanisms of liquefaction and pyrolysis reactions of biomass. Energy Convers. Manage..

[31] Vejerano E, Lomnicki SM, Dellinger B (2012). Formation and stabilization of combustion-generated, environmentally persistent radicals on Ni(II)O supported on a silica surface. Environ. Sci. Technol..

[32] Cheng FC, Jen JF, Tsai TH (2014). Hydroxyl radical in living systems and its separation methods. J. Chromatogr. B.

