# Supplementary material for: Assessing the effect on the generation of environmentally persistent free radicals in hydrothermal carbonization of sewage sludge
Source: Sci Rep. 2019 Nov 19;9:17092. doi: 10.1038/s41598-019-53781-3 (PMC6863856; doi:10.1038/s41598-019-53781-3)
Supplement: Supplementary file 1 — Supplementary information [file 41598_2019_53781_MOESM1_ESM.pdf]

Assessing the effect on the generation of environmentally persistent free radicals in hydrothermal carbonization of sewage sludge

Yuhan Zhu, Jia Wei [*], Yitao Liu, Xiaohui Liu, Jun Li, Jing Zhang

College of Architecture Engineering, Beijing University of Technology,

100 Pingleyuan, Chaoyang district, Beijing 100124, China.

[*]Corresponding author: Jia Wei

E-mail addresses: weij@bjut.edu.cn (Jia Wei)

Samples	Peak area
Liquid separated from the mixture of hydrothermal carbonization	0.0842
Liquid separated from the mixture of hydrochar washing water	0.0838
Unhydrolyzed SS	3.0987
$SHC_{180-2-7}$	34.5786

Table S1 The peak area of samples.

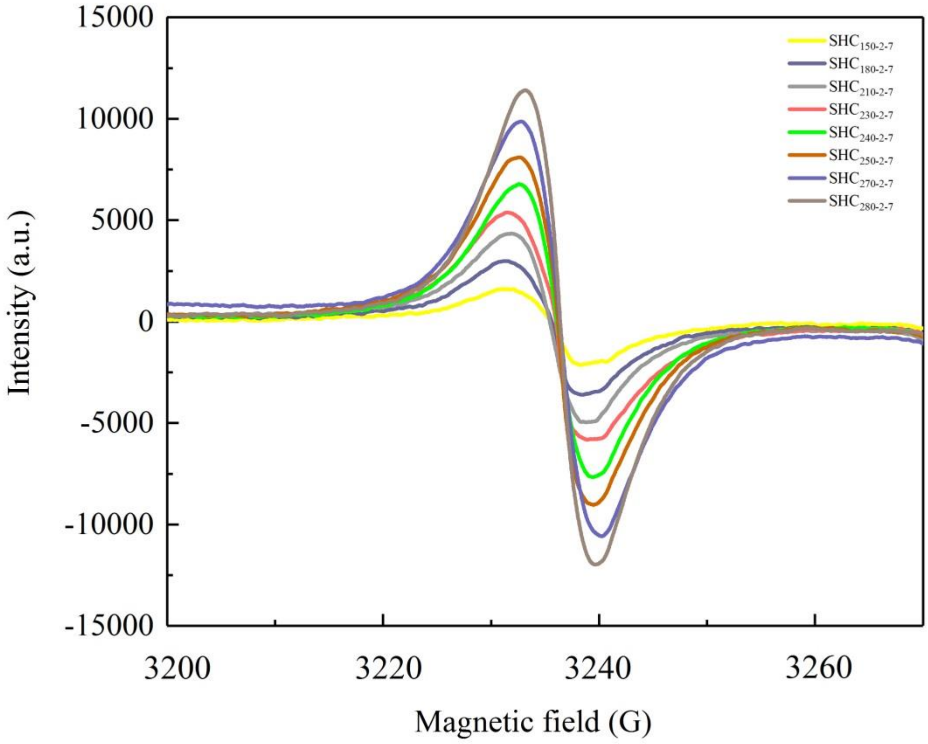

Figure S1. The EPR spectra of SHC prepared at different temperatures.

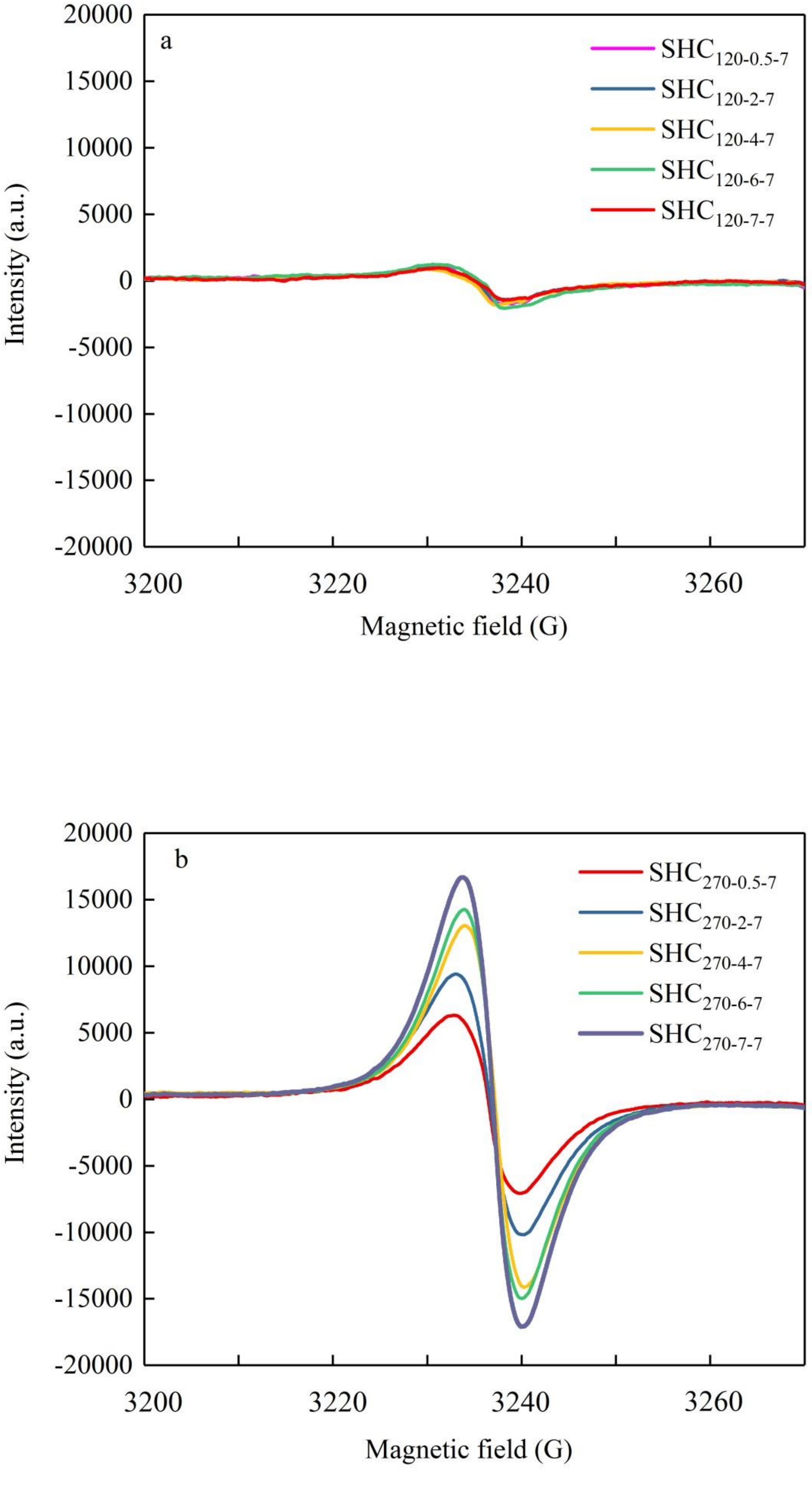

Figure S2. The EPR spectra of the SHC prepared at 120 °C (a) and 270 °C (b)

under different residence times.

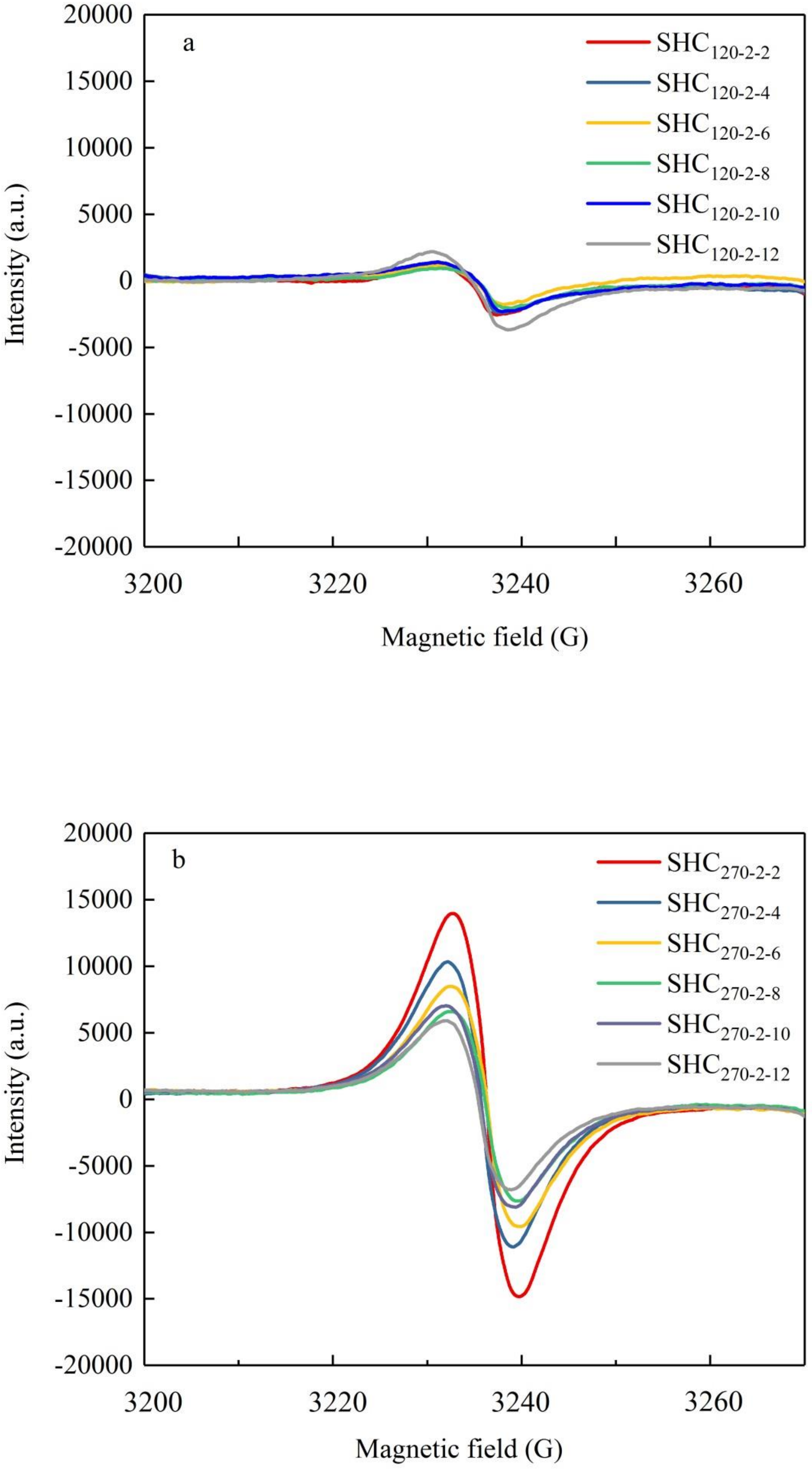

Figure S3. The EPR spectra of the SHC prepared at 120 °C (a) and 270 °C (b) under

different pH.
